# Adaptation of prelimbic cortex mediated by IL-6/STAT3/Acp5 pathway contributes to the comorbidity of neuropathic pain and depression in rats

**DOI:** 10.1186/s12974-022-02503-0

**Published:** 2022-06-11

**Authors:** Yu-Ting Zhao, Jie Deng, He-Ming Liu, Jia-You Wei, Hai-Ting Fan, Meng Liu, Ting Xu, Ting-Feng Chen, Jing-Yi He, Wei-Ming Sun, Tao-Yu Jia, Xue-Qin Zhang, Wen-Jun Xin

**Affiliations:** 1 Neuroscience Program, Zhongshan School of Medicine, The Fifth Affiliated Hospital, Sun Yat-Sen University, Zhongshan Rd. 2, Guangzhou, China; 2 Zhongshan Medical School and Guangdong Province Key Laboratory of Brain Function and Disease, Sun Yat-Sen University, 510080 Guangzhou, China; 3 Department of Interventional Medicine, Guangdong Provincial Engineering Research Center of Molecular Imaging, Guangdong Provincial Key Laboratory of Biomedical Imaging, Sun Yat-Sen University, Guangzhou, China; 4 Guangzhou First People’s Hospital, Guangzhou, China; 5 Department of Applied Psychology, The Affiliated Brain Hospital of Guangzhou Medical University, Xinzao Road, Panyu District, Guangzhou, China; 6 China Center for Brain Science and Brain-Inspired Intelligence, Guangdong-Hong Kong-Macao Greater Bay Area, Guangzhou, China

**Keywords:** Comorbid, Depression, Neuropathic pain, Spared nerve injury, Prelimbic cortex, IL-6

## Abstract

**Background:**

The adaption of brain region is fundamental to the development and maintenance of nervous system disorders. The prelimbic cortex (PrL) participates in the affective components of the pain sensation. However, whether and how the adaptation of PrL contributes to the comorbidity of neuropathic pain and depression are unknown.

**Methods:**

Using resting-state functional magnetic resonance imaging (rs-fMRI), genetic knockdown or overexpression, we systematically investigated the activity of PrL region in the pathogenesis of neuropathic pain/depression comorbid using the combined approaches of immunohistochemistry, electrophysiology, and behavior.

**Results:**

The activity of PrL and the excitability of pyramidal neurons were decreased, and the osteoclastic tartrate-resistant acid phosphatase 5 (Acp5) expression in PrL neurons was upregulated following the acquisition of spared nerve injury (SNI)-induced comorbidity. Genetic knockdown of Acp5 in pyramidal neurons, but not parvalbumin (PV) neurons or somatostatin (SST) neurons, attenuated the decrease of spike number, depression-like behavior and mechanical allodynia in comorbidity rats. Overexpression of Acp5 in PrL pyramidal neurons decreased the spike number and induced the comorbid-like behavior in naïve rats. Moreover, the expression of interleukin-6 (IL-6), phosphorylated STAT3 (p-STAT3) and acetylated histone H3 (Ac-H3) were significantly increased following the acquisition of comorbidity in rats. Increased binding of STAT3 to the Acp5 gene promoter and the interaction between STAT3 and p300 enhanced acetylation of histone H3 and facilitated the transcription of Acp5 in PrL in the modeled rodents. Inhibition of IL-6/STAT3 pathway prevented the Acp5 upregulation and attenuated the comorbid-like behaviors in rats.

**Conclusions:**

These data suggest that the adaptation of PrL mediated by IL-6/STAT3/Acp5 pathway contributed to the comorbidity of neuropathic pain/depression induced by SNI.

## Background

Epidemiological investigation indicated that about 40% patients with neuropathic pain experience psychiatric disorders including anxiety and major depression [1]. Furthermore, the average prevalence of pain in major depression patient is more than 50% [2]. Accumulative evidences have showed that nerve injury, such as spare nerve injure (SNI)-induced neuropathic pain and depression, and some studies proposed that chronic pain and depression may be share common pathological mechanisms but are independent diseases without cause interaction [3, 4]. The reciprocal reinforcement and influence between neuropathic pain and depression substantially escalates the dilemma on the treatment in clinical scenarios [5], which undoubtedly highlights the importance to deliberately dissect the pathogenic mechanisms of comorbidity in the pertinent neuropsychological settings.

Imaging evidence shows that chronic harmful stimulation via changing the intrinsic activity of various brain areas participated in the development and maintenance of nervous system diseases, such as neuropathic pain or major depression [6, 7]. For example, compared with the normal control group, late-onset depression patients have widespread abnormalities in intrinsic brain activity [8]. Among all brain regions, the medial prefrontal cortex (mPFC) plays a hub role for the development of chronic pain and psychiatric disorders [9]. For example, electrophysiological and fMRI (functional magnetic resonance imaging) studies showed the deactivation of mPFC neurons during the perception of pain [10, 11], and chronic stress exposure decreased spine density and dendrite complexity of mPFC neurons, which mediated the occurrence of depression [12, 13]. Furthermore, affective and cognitive components of pain sensations are processed in mPFC [14]. Studies showed that the prelimbic cortex (PrL), as an important subregion of mPFC, is mainly composed of pyramidal neurons [15]. It receives the nociceptive information and is associated with the processing of emotion and working memory [16, 17]. However, whether long term harmful stimulation such as spared nerve injury (SNI) can change intrinsic activity of PrL and contributed to the comorbidity of neuropathic pain and depression are largely unknown.

Although the exact etiology of depression and neuropathic pain is still unclear, relevant data from both animal models and clinical research suggests that the imbalance of proinflammatory cytokines and anti-inflammatory cytokines implicated in the pathophysiology of depression and neuropathic pain [18–20]. For instance, nerve injury significantly increased the expression of proinflammatory cytokine, such as IL-6 and tumor necrosis factor-α (TNF-α) in the nervous system [21], and intrathecal injection interleukin-1β (IL-1β) leads to mechanical allodynia [22]. In addition, early studies showed the positive correlations between the activation of immune system and the development of depression [23], and subsequent clinical studies showed that cytokine interferon-a (IFN-a) therapy significantly reduced the depressive symptoms in patients [24]. Moreover, in some cases, antidepressants have also been reported to reduce proinflammatory cytokine profiles in depressed patients and attenuated the chronic pain [25, 26]. However, whether or how the inflammation cytokine in PrL be involved in the nerve injury-induced neuropathic pain and depression-like symptoms in rats are currently unclear.

Amplitude of low-frequency fluctuation (ALFF) of resting-state functional magnetic resonance imaging (fMRI) reflected the local intrinsic spontaneous activity of resting state in brain [27]. In the present study, we established a rat comorbidity model of neuropathic pain and depression-like behavior on weeks 5 following SNI, and observed the intrinsic spontaneous activity of PrL by examining the change of ALFF in this model. Furthermore, we explored the role of IL-6/STAT3/Acp5 pathway and dissected the mechanism underlying Acp5 upregulation in PrL in the SNI-induced comorbid settings.

## Methods

### Animals and SNI model

Adult male Sprague–Dawley (SD) rats (6 weeks of age, 160–200 g) were obtained from the Institute of Experimental Animals of Sun Yat-Sen University, and were housed individually with access to food and water ad libitum in a room maintained on a 12 h/12 h light/dark cycle. The temperature and humidity were kept at 24 ±  ℃ and 50–60%, respectively. All experimental procedures were approved by the Local Animal Care Committee and were performed in accordance with the guidelines of the National Institutes of Health on animal care and the ethical guidelines. Efforts were made to minimize the suffering and the number of rats used.

Spared nerve injury (SNI) was performed as described by Decosterd and Woolf [28]. Under isoflurane (4%) anesthesia, three peripheral branches of the sciatic nerve of the left hind limb were exposed. The common peroneal and the tibial nerves were ligated and cut (2 mm sections removed), and the sural nerve was kept intact. After that, the surgical incision was sutured in two layers. For the sham procedure, three peripheral branches of the sciatic nerve were exposed without any nerve damage.

### Injection of adeno-associated virus (AAV)

All recombinant adeno-associated virus was purchased from BrainVTA Technology Corp., Ltd or Obio Technology Corp., Ltd. For virus injection, rats were anaesthetized with 4% isoflurane and placed on a stereotaxic frame [29]. The 10 μl Hamilton syringe with glass pipettes was mounted into a micro-infusion pump. Once the target injection site was reached, the injection speed was adjusted to the 25 nl/min under the control of a micro-infusion pump. The pipette was kept in place for an additional 10 min after injection. The location of injections for PrL was determined by the stereotaxic coordinates (AP, + 3.0 mm; ML, ± 0.5 mm; DV, − 4.0 mm) with a minor modification according to different body weights [30], which was histologically confirmed afterward. 150 nl mixture of AAV–EF1a–DIO–Acp5–shRNA–mCherry and AAV–CaMKIIa–Cre, AAV–PV–Cre or AAV–SST–Cre was injected to knockdown the expression of Acp5 in different neurons. To induce the overexpression of Acp5 in pyramidal neurons, the AAV–EF1a–DIO–Acp5–EGFP–WPRE was injected into the PrL together with AAV–CaMKIIa–Cre. For the IL-6R knockdown in PrL pyramidal neurons, 150 nl mixture of AAV–CMV–DIO–IL-6R–shRNA–mEGFP and AAV–CaMKIIa–Cre was injected into PrL.

### Mechanical allodynia

Von Frey hairs were used to assess the 50% withdrawal threshold according to our previously described method [31]. Briefly, each animal was allowed to adaption to a plastic box for 3 consecutive days (15 min/day) before testing. Von Frey filaments with different bending forces were applied alternately to the midplantar surface of hind paw. In the absence of a paw withdrawal response to the initially selected hair, a stronger stimulus was presented afterwards; in the event of paw withdrawal, the next weaker stimulus was then applied. Optimal threshold calculation by this method requires six responses in the immediate vicinity of the 50% threshold.

### Forced swimming test (FST)

FST, as a key feature of depression, is a test for behavioral despair. The protocol of rats’ FST included pre-test stage and the test stage. In the pre-test stage, the rats were taken from their home cage and placed individually in a glass cylinder (50 cm high, 18 cm in diameter) filled with water (24 ± 1 ℃) to a height of 40 cm for 15 min. Next day, the test stage was performed and the process was recorded by video for 5 min. The video recording was analyzed to calculated the time of immobility. Immobility was defined as the absence of all movement, except that necessary to keep the nose above water. During the test, the rat could not touch the tank bottom or escape. Increased time of immobility indicated depression-like behavior. Change the water after every test to avoid any influence on the next test. Among all behavioral tests, the FST was generally arranged as the last one.

### Open field test (OFT)

The open-field test (OFT) was utilized to assess the anxiety-like exploratory and locomotor behaviors. In the present study, the OFT was performed as described previously with a minor adjustment [32]. Briefly, the apparatus consisted of a square 100 cm × 100 cm area with 40 cm high walls and was divided into the central area and outer area. First, the rats in the home cages were removed from their housing room into the testing room for 60 min of acclimation prior to starting the test. The rats were taken from their home cage and gently placed into the center of the area. The process of test was recorded by a digital video camera in a brightly environment. For anxiety-like exploratory behavior, the testing time was 5 min. The traveling distance in the central area and that in the outer area were measured for statistics. For locomotor activity, the testing time was 15 min and the total distance was examined. The activity behaviors were analyzed by software (Shanghai Jiliang Software Technology, Co., Ltd.). The chamber was cleaned with a 75% Ethanol at the end of every test.

### Elevated plus maze (EPM)

Elevated plus maze is a widely used and effective assay for the assessment of depression/anxiety-like behaviors in rodents [33]. Elevated plus maze test was performed as described previously [34]. Briefly, the EPM test consists of four elevated arms of 50 cm long and 10 cm wide. Two closed arms are equipped with 40-cm-high walls and the other two arms are open to the surroundings (open arm). The apparatus is elevated 55 cm above floor. For testing, the rats in their home cage were transferred to the experimental room 30 min prior to the experiment. Then, a rat was placed at the intersection of the four arms of the EPM facing the open arm. The process of test was recorded by a video tracking system with a computer interface for 5 min. The proportion of time spent in the open arms or the closed arms (the time spent in the open or closed arms/5 min) and the number of entries into the open or closed arms were calculated. The maze was rinsed between sessions with 75% alcohol.

### MRI scan and data analysis

Imaging data were collected on a 9.4T animal MRI scanner (Bruker Biospin GmbH, Germany) at the Fifth Affiliated Hospital of Sun Yat-Sen University, Zhuhai, China. The resting-state BOLD signals were collected in the rats with respiratory rate monitored and maintained at 70 times/min after inhalation anesthesia with isoflurane.

During imaging scan, anatomical images were first acquired with the following parameters: repetition time (TR) = 5081.564 ms, echo time (TE) = 21.59 ms, matrix size = 150 × 105, field of view (FOV) = 3.0 × 2.1 cm, slice number = 70, slice thickness = 0.4 mm, slice gap = 0, and resolution = 0.20 × 0.20 × 0.40 mm. For resting-state fMRI scans, an echo-planar image (EPI) sequence with the following parameters was used. TR = 2000 ms, TE = 10.332 ms, flip angle = 90°, matrix size = 100 × 70, field of view (FOV) = 3.0 × 2.1 cm, slice number = 45, slice thickness = 0.6 mm, slice gap = 0, and resolution = 0.30 × 0.30 × 0.60 mm.

The resting-state MRI data were processed using the SPM12 software (Statistical Parametric Mapping 12; http://www.fil.ion.ucl.ac.uk/spm). The first 5 volumes of each fMRI scan were removed to ensure steady-state longitudinal magnetization. The remaining volumes were processed using the following steps: voxel magnification, slice timing correction, realignment, origin correction and coregistration to echoplanar imaging (EPI) templates before resliced at a resolution of 3 × 3 × 3 mm, spatial smoothing using Gaussian kernel with full width half-maximum (FWHM) 6 mm and linear detrending. rsfMRI volumes with FD (Relative framewise displacement) > 0.3 mm were excluded [35].

The fALFF (fractional amplitude of low-frequency fluctuations) values were calculated on detrended data using the DPABI software (https://rfmri.org/dpabi). The ratios of power in the 0.01–0.08 Hz frequency range was calculated by that across the full frequency range (0–0.25 Hz). Then the fALFF values were *z*-transformed prior to statistical analyses [36, 37]. The fALFF results of resting-state fMRI were considered on the voxel-level threshold *p* < 0.05 at the whole-brain level and the cluster-extent threshold were 5 voxels.

### Western blot

Rats brain was immediately removed and sectioned in cold oxygenated artificial cerebrospinal fluid after application of sodium pentobarbital at 50 mg/kg dose (i.p.). The PrL tissues were punched using a 15-gauge cannula and homogenized in Tris containing the inhibitors of proteinase and phosphatase on ice. Proteins were separated by gel electrophoresis SDS–PAGE and transferred to a PVDF membrane. The PVDF membrane was then incubated with primary antibodies against Acp5 (Abcam, 1:1000), IL-6 (CST, 1:1000), p-STAT3 (CST, 1:1000), acetylated histone H3 (K9) (Abcam, 1:1000), acetylated histone H4 (CST, 1:1000), STAT3 (CST, 1:1000), actin (CST, 1:1000) or GAPDH (Abcam, 1:1000) overnight at 4 ℃. The blots were then incubated with secondary antibodies conjugated to horseradish peroxidase. The immunostained bands were acquired by a computer-assisted chemiluminescence imaging analysis system (Tanon 5200).

### RNA extraction and quantitative polymerase chain reaction

Trizol was used to extract total DNA, and the reverse transcription was performed following the protocol of polymerase chain reaction (PCR) production kit (Accurate Biology, AG 11706). Table 1 shows the primers sequences of the investigated mRNA for PCR assay. The reaction cycle conditions are as follows: an initial denaturation at 95 °C for 3 min, followed by 40 thermal of 10 s at 95 °C, 20 s at 58 °C, and 10 s at 72 °C. The ratio of mRNA expression in the PrL tissues was analyzed by the 2^−**△△**CT^ method.

**Table 1:** Specific primer sequences

Gene	Primer	Sequence
S100a9 (rat)	Forward	5′-AGACATCATGGAGGACCTGGACAC-3′
	Reverse	5′-TGGGTTGTTCTCATGCAGCTTCTC-3′
Ltf (rat)	Forward	5′-CTGCTTGTCAACCAGACCAACTCC-3′
	Reverse	5′-CCGTTCTCGTCACCAATACACAGG-3′
Slpi (rat)	Forward	5′-GTGCGGTACTGACTGGGAATGC-3′
	Reverse	5′-CAGGCTTCTTCTTCACTGGTCCAC-3′
Cd244 (rat)	Forward	5′-ACATCAGAGCACCTGGAGGAGAC-3′
	Reverse	5′-GCAGGAAGAGTGACAACAGGACAG-3′
Acp5 (rat)	Forward	5′-ATGACGCCAATGACAAGAGGTTCC-3′
	Reverse	5′-TTGTGCCGAGACATTGCCAAGG-3′
Areg (rat)	Forward	5′-TTACTTTGGCGAACGGTGTGGAG-3′
	Reverse	5′-GAAGCAGGACGGCGGTAATGAT-3′
Six1 (rat)	Forward	5′-CTCCCTCCTCCTCCTCTTTGTCTTC-3′
	Reverse	5′-TTTTCCTCTTCCCTAAACCGTTTCTCC-3′
β-actin (rat)	Forward	5′-AGGGAAATCGTGCGTGACAT-3′
	Reverse	5′-GAACCGCTCATTGCCGATAG-3′

### Fluorescence in situ hybridization (FISH) and immunofluorescence

Rats were perfused through the ascending aorta with 4% paraformaldehyde under anesthesia. The PrL tissues were cut into 25 µm-thick transverse sections after 30% DEPC–sucrose dehydration at 4 ℃ and hybridized at 42 ℃ for 16 h with the 5ʹ-TYE665-label Acp5 probe 5ʹ-ACGTATCCATCACCAATCTCT-3ʹ (1:200, QIAGEN). The sections were then incubated at 4℃ overnight with primary antibodies against NeuN (Millipore, 1:500), Iba1 (Abcam, 1:400), GFAP (CST, 1:400), CaMKIIa (Abcam, 1:200), PV (Novusbio, 1:400) or SST (ABclonal, 1:100). After that, the sections were incubated with fluorescein isothiocyanate-conjugated secondary antibody at 37 °C for 60 min. The stained sections were examined using with a Nikon confocal microscope equipped.

For IL-6R immunofluorescence, the PrL tissues were cut into 25 µm-thick transverse sections and incubated with primary antibodies against IL-6R (Santa, 1:50), NeuN (Millipore, 1:500), Iba1 (Abcam, 1:400) or GFAP (Abcam, 1:400) at 4 ℃ overnight. After that, the sections were incubated with Cy3 or fluorescein isothiocyanate-conjugated secondary antibody at 37 °C for 60 min. The stained sections were examined using with a Nikon confocal microscope equipped, and images were captured with a Nikon DS-Qi2 camera.

### Cell culture

PC-12 cells were purchased from Guangzhou Xinyuan Technology Co, Ltd., and were cultured in about 6 ml of the RPMI1640 complete medium (RPMI medium1640, 5%FBS, the medium was purchased from Gibco) with 1% penicillin–streptomycin in a 25 cm^2^ breathable cell culture bottle (NEST, China), and subsequently placed in the carbon dioxide incubator containing 5% carbon dioxide and 95% oxygen, at a constant temperature of 37 ℃. After the cells recovered and returned to normal, the PC-12 cells were incubated with 5 ng/ml recombinant rat IL-6 protein (R&D systems). After 12 h, the cells were collected for subsequent experiments.

### Patch-clamp recording in PrL slices

Slices of the rat PrL were prepared as our described previously [38]. Briefly, brain tissue was removed and incubated in oxygenated (95% O_2_–5% CO_2_) cold artificial cerebrospinal fluid (ACSF) containing (in mM): 127 NaCl, 3.1 KCl, 1.2 MgCl_2_, 2.4 CaCl_2_, 26 NaHCO_3_, and 10 glucose, pH 7.3, osmolarity 300–310 mOsm/L. 400 µm thick PrL slices were prepared with a vibratome (DTK-1000). Slices were incubated in gassed ACSF for at least 1 h at 32 °C. Then, an individual slice was transferred to a recording chamber and continually perfused with oxygenated ACSF solution at RT. PrL neurons were visualized using a 60× water-immersion objective on an upright infrared Nikon microscope (Nikon, Tokyo, Japan). The neurons in lamina III/IV of the PrL were recorded using pipettes containing an internal solution (135 mM k-gluconate, 0.5 mM CaCl_2_, 2 mM MgCl_2_, 5 mM EGTA, 5 mM HEPES, 5 mM Mg-ATP, and 0.5% biocytin, pH 7.3). Action potentials (Aps) were evoked by current injection every 10 s with step intervals of 20 pA from − 20 to 180 pA over a period of 400 ms. The relationship between firing frequency and injected current was analyzed using Clampfit10.4 (Axon Instruments Inc., USA).

### Chromatin immunoprecipitation assays

Commercial kit (CST) was used to perform chromatin immunoprecipitation (ChIP) assays. The PrL tissues or PC12 was collected and placed in 1% formaldehyde for 10 min at room temperature to crosslink target protein with chromatin. The formaldehyde was then inactivated by addition of 125 mM glycine. Sonicated chromatin extracts containing DNA fragments were immunoprecipitated using antibodies against p-STAT3 or Ac-H3 and pre-blocked protein G-sepharose beads overnight at 4 ℃. The next day, the chromatin–protein–antibody–bead complexes were eluted, and the DNA was extracted. The precipitated DNA was resuspended in nuclease-free water, and qPCR was performed. Primers 5ʹ-TGGGGTGTGCCTTCTGGA-3ʹ and 5ʹ-AGTTGTGTATTTGAAGTCA-3ʹ were projected to amplify a 139 bp fragment (− 1389/− 1250) sequence, which was localized on the Acp5 promoter. Finally, the ratio of ChIP/input in the PrL was calculated.

### Co-immunoprecipitation (Co-IP)

Co-IP was conducted using a Co-Immunoprecipitation Kit (Pierce). PrL tissues were excised quickly and placed in lysis buffer. A Pierce Spin Column was placed in a microcentrifuge tube. After addition of AminoLink Plus Coupling Resin and affinity-purified p-STAT3 antibody (CST, 1:100) or P300 antibody (Abcam, 10 μg), the complex was incubated on a rotator at room temperature for 90–120 min to ensure antibody immobilization. Tissue lysates were added to the appropriate resin columns and incubated with gentle rocking overnight at 4 °C. The spin columns were then centrifuged and placed in new collection tubes, elution buffer was added, and the flow-through was collected by centrifugation. The immune complexes in the flow-through were analyzed by western blotting using P300 antibody or p-STAT3 antibody. All co-IP steps were performed at 4 °C unless otherwise indicated.

### Statistical analyses

SPSS 25.0 was used to analyze the data; the results are shown as the mean ± s.e.m. The data were analyzed using the two independent samples *t* test or one-way ANOVA followed by Dunnett’s T3 or Tukey’s post hoc test. When tests of normality were not satisfied, the permutation test was substituted. The criterion of statistical significance was *p* < 0.05. Although no power analysis was performed, the sample size was determined according to previous publications in behavioral and pertinent molecular studies. All measurements were taken from distinct samples.

## Results

### SNI induced the comorbidity of neuropathic pain and depression in rats

**Fig. 1:**
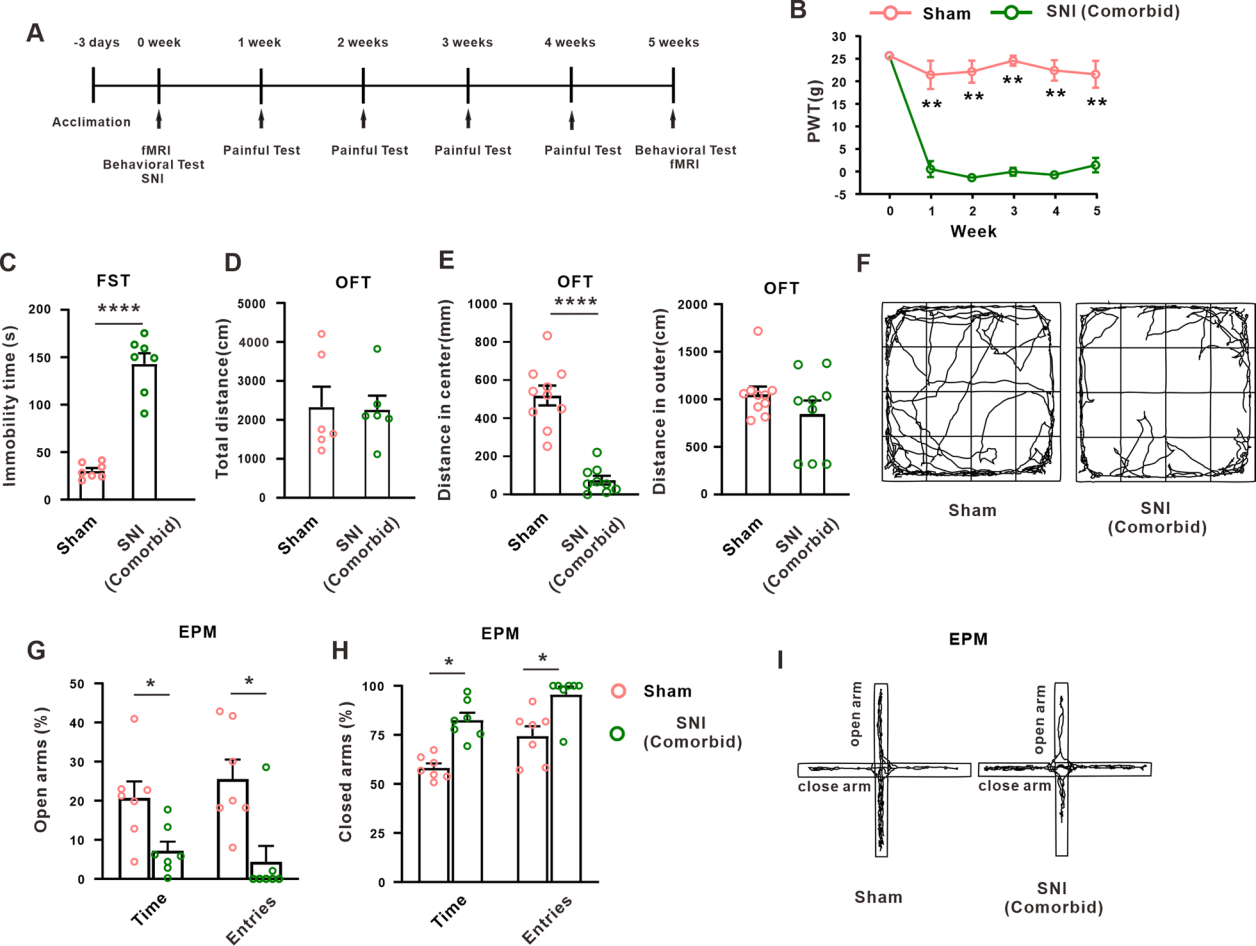
SNI induced the mechanical allodynia and depression-like behaviors in rats. **A** Schematic timeline of the experimental procedure. **B** Paw withdrawal threshold on the ipsilateral hind paw was significantly decreased following SNI treatment in rats (*n* = 7 in each group, paired *t*-test, **p < 0.01 versus the sham group). **C** Immobility time of the FST was increased on weeks 5 after SNI (*n* = 7 in each group, unpaired *t*-test, ****p < 0.0001 versus the sham group). **D** Total distance of rats was examined in OFT for 15 min following SNI (*n* = 6 in each group, unpaired *t*-test, *p* = 0.9137 versus the sham group). **E** Distance of central zone (left) and outer zone (right) were examined in OFT for 5 min following SNI (*n* = 9 or 10 in each group, unpaired *t*-test, ****p < 0.0001 versus the sham group). **F** Representative rat trajectory map in the OFT. **G** Percentage of time and the percentage of entries in the open arms was significantly decreased in EPM on 5 weeks after SNI (*n* = 7 in each group, unpaired *t*-test, for time: *p = 0.0159 versus the sham group; for entries: **p = 0.0053 versus the sham group). **H** Percentage of time and the percentage of entries in the closed arms was examined in EPM following SNI (*n* = 7 in each group, unpaired *t*-test, for time: ***p = 0.0001 versus the sham group; for entries: *p = 0.0172 versus the sham group). **I** Representative trajectory map of EPM test for rats. Data are expressed as mean ± SEM

In the present study, we performed the spared nerve injury (SNI) in rats, and observed the change of mechanical allodynia and depression-like behavior on 5 weeks later (Fig. 1A). In the mechanical withdrawal threshold test, the SNI rats exhibited significant mechanical allodynia on week 1 and maintained to the end of the experiment (weeks 5) (Fig. 1B). In the depression-like behavioral test, the SNI rats showed the increased immobility time in forced swimming test (FST) on weeks 5 compared with the sham group (Fig. 1C). Since the patients with depression often experiences anxiety-like manifestation, we further explored the anxiety-like behavior using the open field test (OFT) and the elevated plus maze (EPM). In the OFT, the total travel distance of 15 min between weeks 5 and the sham group was similar (Fig. 1D), suggest that the locomotor activity was not affected on weeks 5 following SNI. Importantly, the travel distance in central zone (Fig. 1E, right), but not in outer zone (Fig. 1E, left), was significantly decreased on weeks 5 in SNI rats. In the EPM test, compared with the sham group, the percentage of spent time and the percentage of entries in the open arms was significantly decreased (Fig. 1G), and the percentage of spent time and entries in the closed arms was increased on weeks 5 after SNI (Fig. 1H). These results suggested the establishment of neuropathic pain/depression comorbid on 5 weeks following SNI in rats, and raised a hypothesis that neuropathic pain and depression-like behavior may share the similar mechanism following SNI.

### Decreased activity of prelimbic cortex was involved in the comorbidity of neuropathic pain and depression in rats

**Fig. 2:**
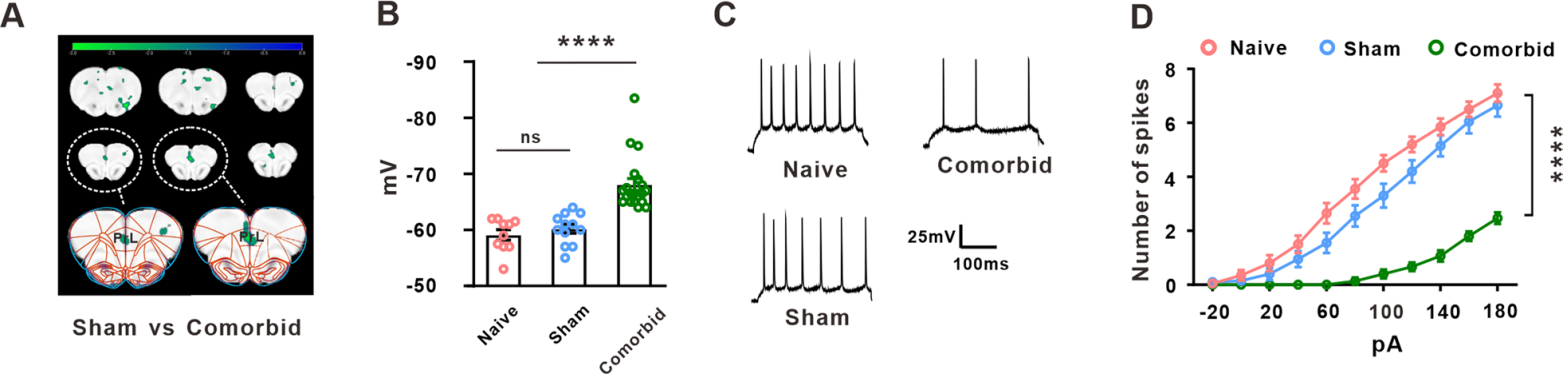
Activity and the excitability of pyramidal neurons in PrL on weeks 5 in the comorbidity of rats. **A** fALFF value in whole brain were examined by rs-fMRI on weeks 5 following SNI (*n* = 11 in each group). **B** Resting membrane potential of PrL pyramidal neurons was examined in naïve, sham and comorbidity group (*n* = 15 or 20 in each group, One-way ANOVA, F_*(2,47)*_ = 20.93, ****p < 0.0001 versus the sham group). **C** Representative traces of action potential with injected current of 140 pA in both naïve, sham and comorbidity groups. **D** Action potential firing rate of pyramidal neurons was decreased in comorbidity rats (*n* = 15 or 20 in each group, Two-way ANOVA, ****p < 0.0001 versus the sham group). Data are expressed as mean ± SEM

Resting-state fMRI (rsfMRI) is an increasingly popular method of MRI that investigates synchronous (spontaneous) activity of brain regions in the absence of an explicit signal correlation-based task. In the present study, we assessed the adaptive changes in the brain regions of rats between comorbidity group and sham group, and found that the fALFF value of PrL brain region was significantly decreased in comorbidity group in rats (Fig. 2A), indicating a reduced neural activity in PrL. As previous studies showed that the pyramidal neurons of prelimbic cortex (PrL) played an important role in mental disorder, we then observed the excitability of pyramidal neurons using whole-cell recordings. The results revealed that the resting membrane potential (RMP) of comorbidity rats become more negative than that of sham group (Fig. 2B). Moreover, the frequency of action potentials was significant decreased in pyramidal neurons of PrL in rats with the comorbid symptoms compared with the sham group (Fig. 2C, D). These data indicated the decreased activity and excitability of pyramidal neurons in PrL in the rats with comorbidity of neuropathic pain/depression following SNI.

### Acp5 was significantly increased in PrL in the comorbid rats

**Fig. 3:**
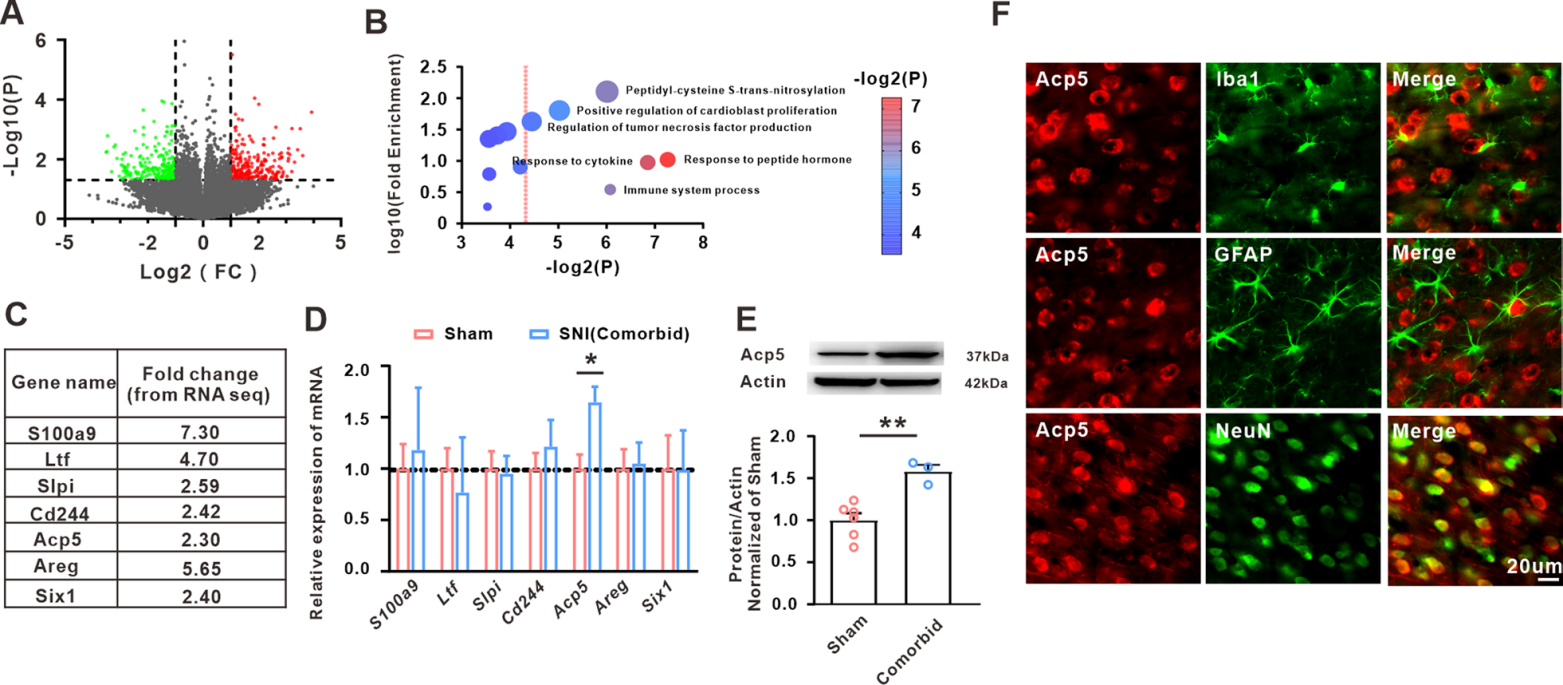
Acp5 expression in the neuropathic pain/depression comorbidity rats. **A** Volcano plots of the changed genes from the GEO database (GSE91396). **B**, **C** From 6 significant GO terms, seven target genes associated with inflammation were selected. **D** mRNA levels of S100a9, Ltf, Slip, Cd244, Acp5, Areg and Six1 were explored in PrL in comorbidity rats (*n* = 3 in each group, unpaired *t*-test, *p = 0.0342 versus the sham group). **E** Protein levels of Acp5 was measured in sham group and comorbid group (*n* = 3 or 6 in each group, unpaired *t*-test, **p = 0.0033 versus the sham group). **F** Immunofluorescence staining of Acp5 (red) colocalized with NeuN (neuron marker, green) but not with GFAP (astrocyte marker, green) or Iba1 (microglial marker, green) (*n* = 2). Data are expressed as mean ± SEM

To explore the potential molecular mechanism of neuropathic pain/depression comorbidity, one RNA-seq profile (GSE91396) from the GEO database was analyzed to identify the changed genes of the mPFC in the SNI-induced depression in mice. A total of 461 genes were obtained with a criterion of *p* < 0.05 and FC ≥ 2 (Fig. 3A). Next, we performed the Gene Ontology (GO) analysis and selected 6 significant terms (Fig. 3B). Studies showed that inflammatory responses was involved in the emotional disorder and neuropathic pain [39, 40]. From these six terms, we selected seven upregulated target genes including *S100a9*, *Ltf*, *Slip*, *Cd244*, *Acp5*, *Areg* and *Six1* (Fig. 3C), which were closely related to inflammatory responses. PCR further identified that only the *Acp*5 mRNA expression of comorbidity rats was significantly upregulated in PrL relative to the sham group (Fig. 3D). Western blot also confirmed that the level of Acp5 protein significantly increased in comorbidity group (Fig. 3E). Furthermore, the results of fluorescence in situ hybridization (FISH) showed that Acp5 mRNA was colocalized with NeuN-positive cells, but not GFAP-positive cells or Iba1-positive cells in PrL (Fig. 3F). The results suggest that comorbidity of neuropathic pain/depression-induced by SNI significantly increased the Acp5 level in the PrL neurons.

### *Acp5* contributed to comorbidity-related behavior through modulating excitability of PrL pyramidal neurons

**Fig. 4:**
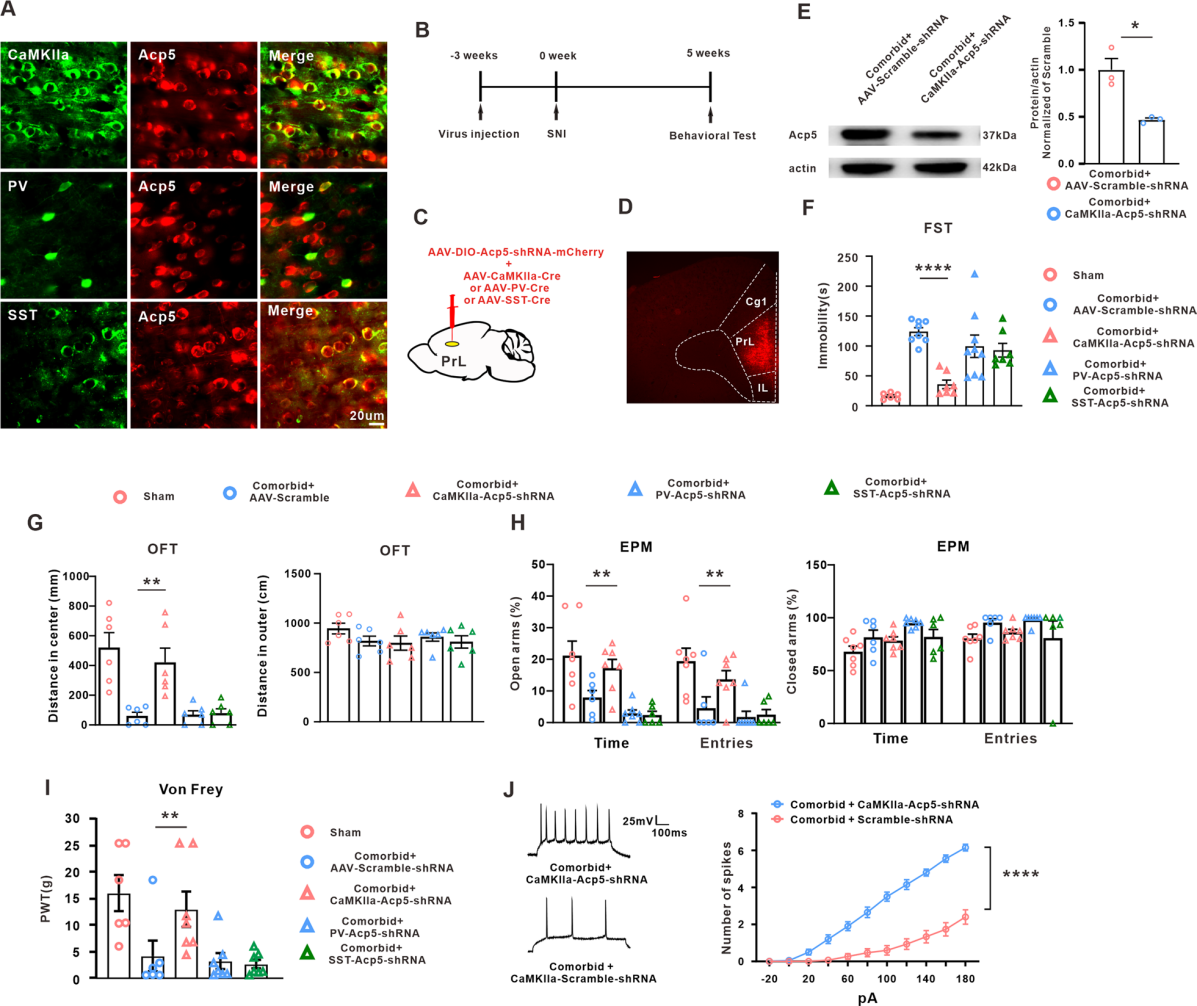
Knockdown of Acp5 in pyramidal neurons alleviated the SNI-induced comorbid-like symptoms. **A** Colocalization of Acp5 and CaMKIIα, PV and SST in PrL (40 × objective, scale bars, 20 µm, *n* = 2). **B** Schematic timeline of the experimental procedure for AAV injection and SNI. **C** Injective schematic of the AAV-Acp5-shRNA together with Cre-dependent virus of different neurons promoter. **D** Typical images of viral expression within PrL region. **E** Intra-PrL microinjection of AAV-Acp5-shRNA significantly decreased the Acp5 expression in the comorbidity rats **(n** = 3 in each group, unpaired *t*-test, *p = 0.0121 versus the scramble group). **F** Immobility time of the FST was decreased by Acp5 knockdown in CaMKII-positive neurons, but not in PV-positive neurons or SST-positive neurons (*n* = 6 or 7 in each group, One-way ANOVA, *F*_(4,32)_ = 13.10, ****p < 0.0001 versus the correspondence scramble group). **G** Distance in central zone (left) and outer zone (right) in OFT were examined following knockdown the Acp5 expression in different type neurons, respectively (*n* = 6 in each group, One-way ANOVA, left: *F*_(4,25)_ = 11.65, ****p < 0.0001 versus the correspondence scramble group; right: *F*_(4,25)_ = 1.419, *p* = 0.2567 versus the correspondence scramble group). **H** Percentage of time and the percentage of entries in the open arms (left) and the closed arms (right) was measured in the EPM test by knockdown Acp5 expression in different neurons of PrL (*n* = 6 or 7 in each group, One-way ANOVA, left: for time, *F*_(4,28)_ = 9.476, ****p < 0.0001 versus the correspondence scramble group; for entries, *F*_(4,28)_ = 7.190, ***p = 0.0004 versus the correspondence scramble group). **I** Application AAV-DIO-Acp5-shRNA together with AAV-CaMKIIa-Cre attenuated the mechanical allodynia (*n* = 6 or 7 in each group, One-way ANOVA, *F*_(4,28)_ = 5.901, **p = 0.0014 versus the correspondence scramble group). **J** Knockdown of Acp5 in pyramidal neurons prevented the decrease of action potential number (*n* = 15 or 20 in each group, Two-way ANOVA, ****p < 0.0001 versus the correspondence scramble group). Data are expressed as mean ± SEM

It is well known that there primarily exist CamkIIa-positive pyramidal neurons, Parvalbumin-positive (PV-positive) neurons and Somatostatin-positive (SST-positive) neurons in PrL. FISH was used to explicit whether Acp5 expression was limited to specific neurons in PrL. The results showed that Acp5 was present in both excitatory (CaMKIIa-positive neurons) and inhibitory neurons (PV- and SST-positive neurons) in PrL (Fig. 4A). To determine which type of neurons contributes to the comorbidity, we designed an adeno-associated virus (AAV) carrying Cre-dependent Acp5–shRNA, and injected the virus together with three different AAV–Cre with characteristic neuronal promoter into the bilateral PrL, respectively (Fig. 4B–D). An obvious decrease of the Acp5 level in comorbid rats following AAV–CaMKII–Acp5–shRNA injection suggested the efficiency of the AAV virus (Fig. 4E). Importantly, behavioral test showed that specific knockdown of *Acp5* in pyramidal neurons, but not in PV neurons or SST neurons, significantly alleviated depression-like behavior, including the reduced immobility time in FST (Fig. 4F), the increased center distance in OFT (Fig. 4G) and the spent time and entries in open arms in EPM test (Fig. 4H). Clinically, antidepressant drug, such as clomipramine exerted an analgesic effect [41], we observed the effect of *Acp5* knockdown on mechanical allodynia. The result showed that knockdown Acp5 expression in pyramidal neurons, but not in PV- or SST-neurons, increased paw withdrawal threshold in the comorbidity rats (Fig. 4I). Moreover, electrophysiological results showed that *Acp5* knockdown significantly increased the number of action potentials in pyramidal neurons in the comorbidity group (Fig. 4J).

**Fig. 5:**
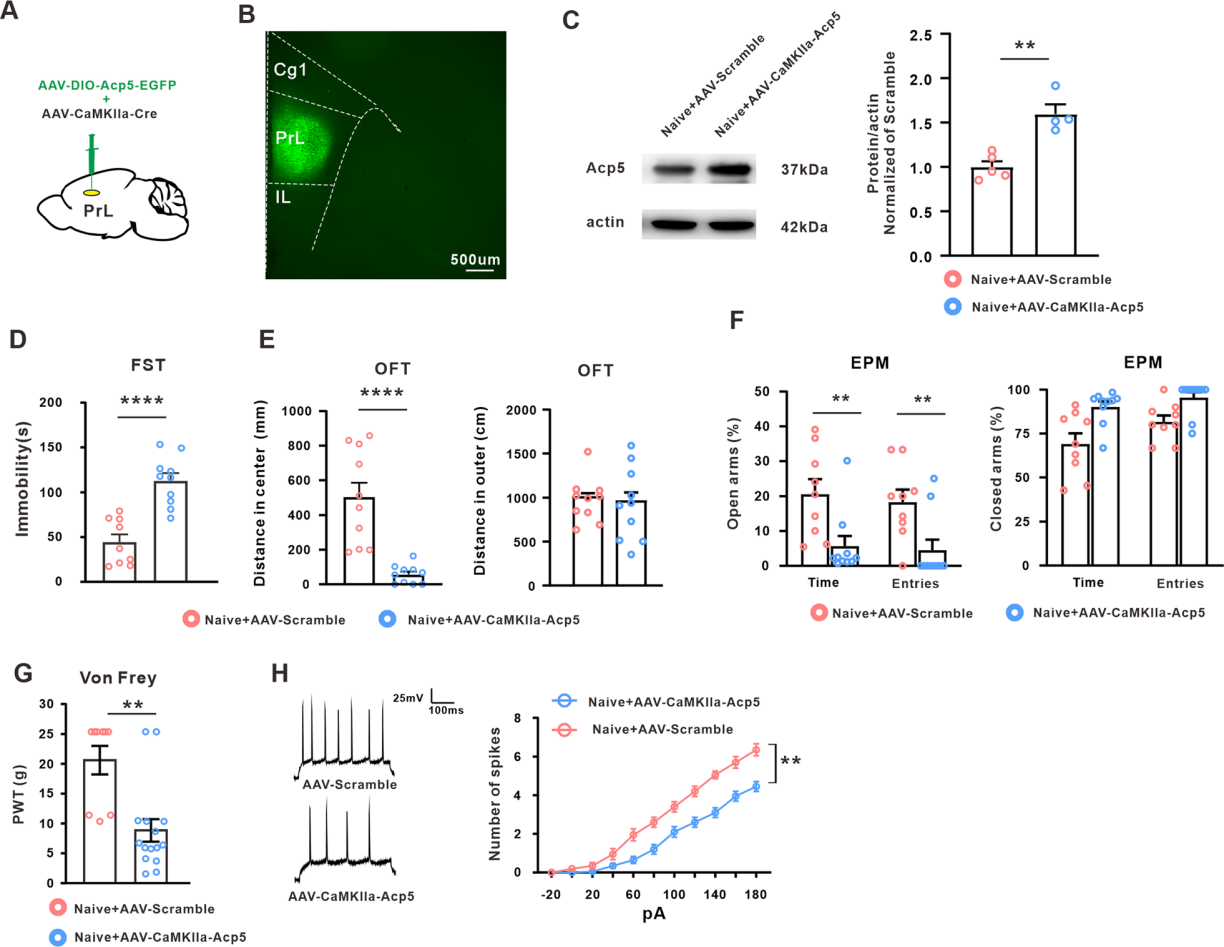
Overexpression of Acp5 in PrL pyramidal neurons induced the comorbid-like behavior in naïve rats. **A** Injective schematic of the AAV-DIO-Acp5-EGFP together with AAV-CaMKIIa-Cre. **B** Typical images of viral expression within PrL region (Scale bar, 500 µm). **C** Intra-PrL injection of AAV-Acp5 significantly increased the Acp5 expression in naïve rats (*n* = 4 or 5 in each group, unpaired *t*-test, **p = 0.0017 versus the scramble group). **D** Overexpression of Acp5 in PrL pyramidal neurons increased the immobility time in the FST. (*n* = 9 or 10 in each group, unpaired *t* test, ****p < 0.0001 versus the correspondence scramble group). **E** Distance in central zone and outer zone in open field test were examined following the intra-PrL injection of AAV-Acp5 (*n* = 10 in each group, unpaired *t* test, ****p < 0.0001 versus the correspondence scramble group). **F** Time spent and entries number in the open arms and the closed arms was measured in the EPM test following overexpression of Acp5 in PrL pyramidal neurons. (*n* = 9 or 10 in each group, unpaired *t* test, for time, **p = 0.0085 versus the correspondence scramble group; for entries, **p = 0.0086 versus the correspondence scramble group). **G** Overexpression of Acp5 in PrL pyramidal neurons induced the mechanical allodynia. (*n* = 9 or 15 in each group, unpaired *t*-test, ***p < 0.0009 versus the correspondence scramble group). **H** Number of action potential in PrL pyramidal neurons was decreased following overexpression of Acp5 in PrL pyramidal neurons in naïve rats (*n* = 20 in each group, Two-way ANOVA, ****p < 0.0001 versus the correspondence scramble group). Data are expressed as mean ± SEM

To further confirm the role of Acp5 in pyramidal neurons of PrL in the comorbidity of neuropathic pain and depression in rats, we overexpressed the Acp5 by bilateral injection of AAV–DIO–Acp5–EGFP into the PrL together with AAV–CaMKIIa–Cre (Fig. 5A, B). Western blot showed that intra-PrL injection AAV–CaMKII–Acp5 indeed increased the Acp5 level on day 21 (Fig. 5C). The behavioral test showed that overexpression of Acp5 in PrL pyramidal neurons induced multiple depression-like behaviors in various assays including FST, OFT and EPM on day 21 after virus injection (Fig. 5D–F), and the rats displayed mechanical allodynia (Fig. 5G). Whole-cell recordings of pyramidal neurons showed a decrease in the spike number in PrL slices from Acp5-overexpressed rats (Fig. 5H). All above results suggest that the increased Acp5 inhibited the pyramidal neurons excitability and contributed to comorbid-like behavior of neuropathic pain and depression induced by SNI in rats.

### IL-6/STAT3 signal pathway contributed to the comorbidity of neuropathic pain and depression

**Fig. 6:**
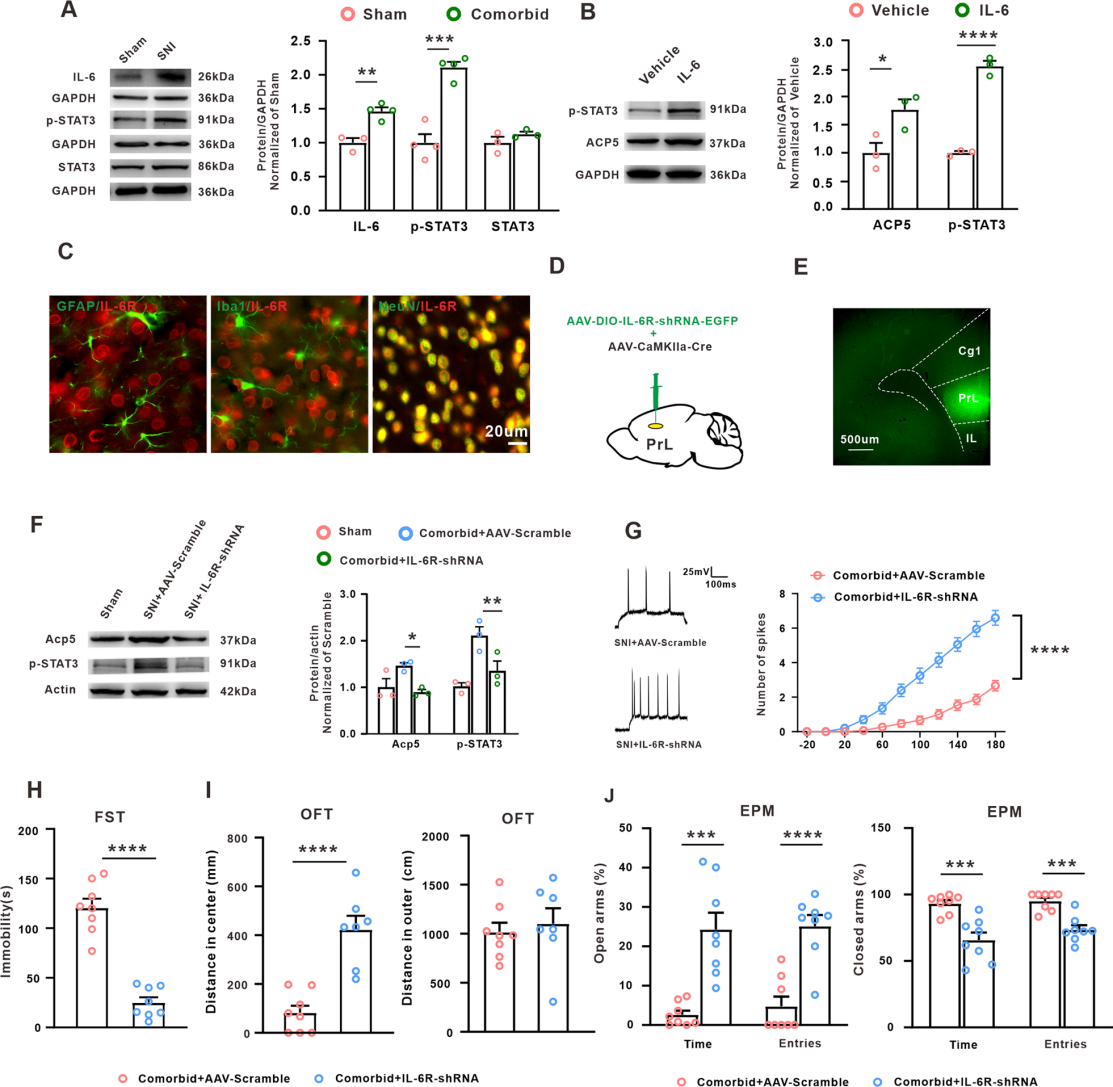
Role of IL-6/STAT3 signal pathway in the comorbidity of neuropathic pain/depression. **A** Expression of IL-6, p-STAT3 and STAT3 in PrL were explored on weeks 5 following the SNI (*n* = 3 or 4, unpaired *t* test, **p = 0.0034, ***p = 0.0003 versus the sham group). **B** Incubation of IL-6 increased the expression of p-STAT3 and Acp5 in PC12 cells (*n* = 3, unpaired *t* test, *p = 0.0384, ****p < 0.0001 versus the vehicle group). **C** IL-6R expression was colocalized with NeuN-positive cells, but not GAFP-positive cells or Iba1-positive cells (scale bars, 20 µm, *n* = 2). **D** Injective schematic of the AAV-Il-6R-shRNA. **E** Representative images of viral expression within PrL region (scale bars, 500 µm). **F** Intra-PrL microinjection AAV-IL-6R-shRNA decreased the p-STAT3 or Acp5 upregulation induced by SNI (*n* = 3, One-way ANOVA, F_*(2,6)*_ = 6.756, *p = 0.0291, F_*(2,6)*_ = 11.03, **p = 0.0098 versus the correspondence scramble group.) **G** Knockdown of IL-6R in PrL pyramidal neurons increased the number of action potential in comorbid group (*n* = 15 or 20 in each group, Two-way ANOVA, ****p < 0.0001 versus the correspondence scramble group). **H** IntraPrL injection of AAV-IL-6R-shRNA decreased the immobility time induced by SNI in the FST (*n* = 8 in each group, unpaired *t*-test, ****p < 0.0001 versus the correspondence scramble group). **I** Distance in the central zone (left) and the outer zone (right) in OFT were examined following the intraPrL injection of AAV-IL-6R-shRNA (*n* = 7 or 8 in each group, unpaired *t* test, ****p < 0.0001 versus the correspondence scramble group). **J** Time spent and entries number in the open arms (left) and the closed arms (right) was measured in the EPM test following knockdown of IL-6R in PrL pyramidal neurons. (*n* = 8 in each group, unpaired *t* test, ***p = 0.0002, ****p < 0.0001 versus the correspondence scramble group). Data are expressed as mean ± SEM

IL-6/STAT3 pathway has been reported to be involved in chronic pain and mental disorder [42, 43], so we tested the role of IL-6/STAT3 pathway in the SNI-induced comorbid-like behavior in rats. We found that the expressions of IL-6 and p-STAT3 were significantly increased in the comorbidity group (Fig. 6A). In PC-12 cells, incubation of IL-6 also upregulated the expressions of p-STAT3 and the Acp5 (Fig. 6B). IL-6 exerts the biological effects by binding with its receptor IL-6R. The present results revealed that IL-6R was colocalized with the NeuN-positive cells, but not the GAFP-positive cells or Iba1-positive cells in PrL region (Fig. 6C). Next, we explored the role of IL-6R and p-STAT3 in PrL pyramidal neurons in the development of the comorbidity. The results showed that microinjection of AAV–DIO–*IL-6R*–shRNA–EGFP together with AAV–CaMKIIa–Cre (Fig. 6D, E) prevented the upregulation of p-STAT3 and Acp5 induced by comorbidity (Fig. 6F). Furthermore, knockdown of IL-6R in PrL pyramidal neurons increased the number of spikes in comorbid rats (Fig. 6G), and the symptom of depression-like behaviors, such as the immobility time in FST (Fig. 6H), the center distance in OFT (Fig. 6I) and the spent time and entries in open arms in EPM (Fig. 6J), was also significantly improved following AAV–IL-6R–shRNA injection. These results suggested that IL-6/STAT3 signal pathway contributed to the comorbidity of neuropathic pain and depression induced by SNI.

### The activated transcription factor STAT3 regulated Acp5 expression following the acquisition of comorbidity of neuropathic pain and depression

**Fig. 7:**
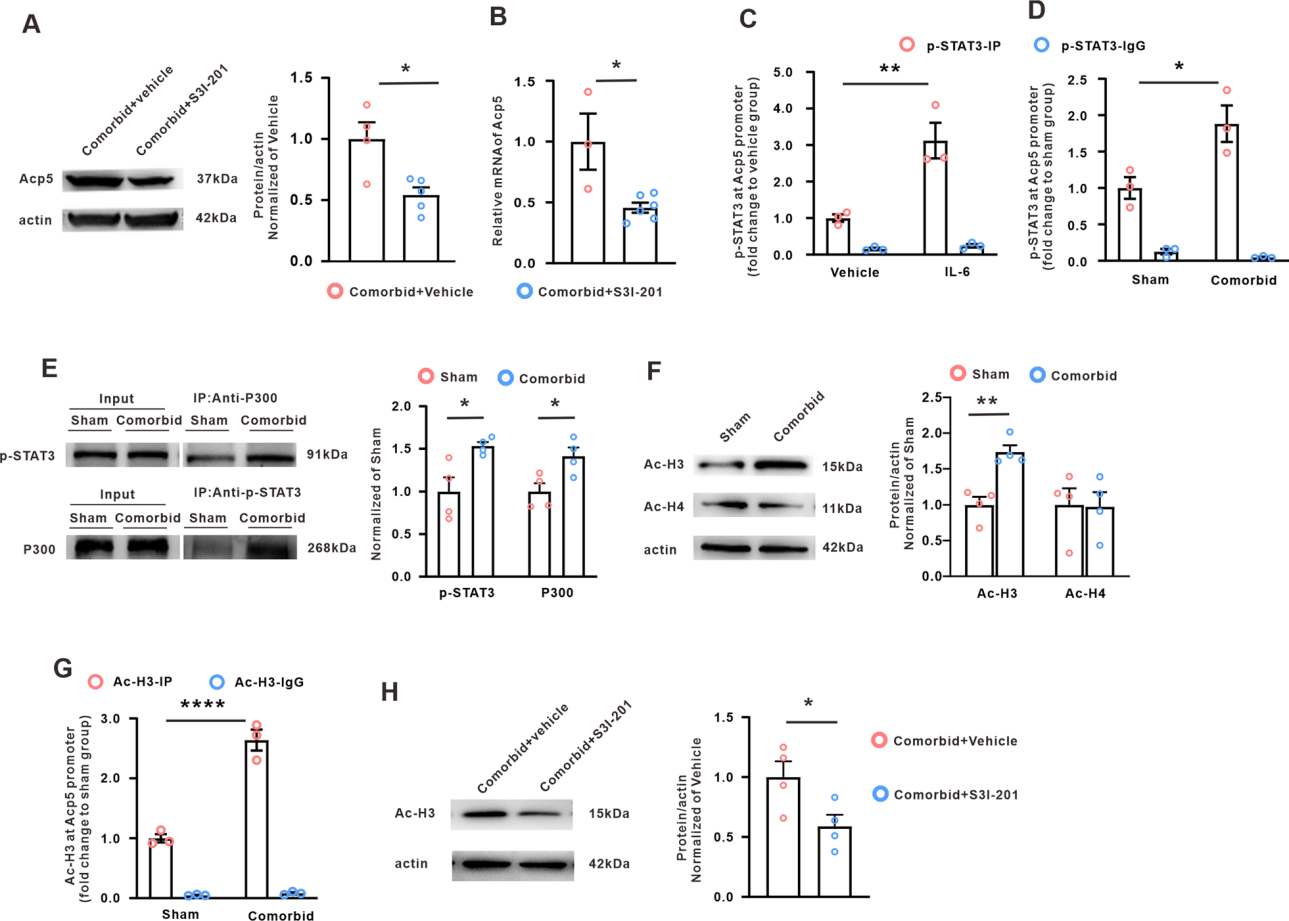
Activated STAT3 by binding p300 increased the level of acetylated histone H3 on the Acp5 promoter. **A**, **B** Intra-Prl injection of S3I-201 prevented the increases of Acp5 proteins and mRNA induced by the acquisition of comorbid following SNI (*n* = 3 or 5, unpaired t test, for protein, *p = 0.0134, for mRNA, *p = 0.0124, versus the correspondence vehicle group). **C** Chromatin immunoprecipitation assay was performed with p-STAT3 antibody following incubation of IL-6 in PC12 cells (*n* = 3 in each group, unpaired *t* test, **p = 0.002 versus the vehicle group). **D** Chromatin immunoprecipitation assay was performed with p-STAT3 antibody in comorbidity rats (*n* = 3 in each group, unpaired *t test*, *p = 0.0119 versus the sham group). **E** The interaction between p-STAT3 and P300 was increased following the acquisition of comorbidity after SNI in rats (*n* = 4 in each group, unpaired *t test*, for p-STAT3, *p = 0.0225, for P300, *p = 0.0279, versus the sham group). **F** The histone acetylation of H3 K9, but not the global histone acetylation of H4, was upregulated on weeks 5 following SNI (*n* = 4 in each group, unpaired *t test*, **p = 0.0025 versus the sham group). **G** Chromatin immunoprecipitation was performed using Ac-H3 antibody on weeks 5 after SNI (*n* = 3 in each group, unpaired *t test*, ****p < 0.0001 versus the sham group). **H** IntraPrL injection of S3I-201 reversed the increased histone acetylation of H3 induced by SNI (*n* = 4 in each group, unpaired *t test*, *p = 0.0459 versus the vehicle group). Data are expressed as mean ± SEM

Next, we observed the role of p-STAT3 in the Acp5 expression following the acquisition of comorbidity of neuropathic pain and depression. The results showed that intra-PrL injection of STAT3 inhibitor S3I-201 inhibited the increase of Acp5 protein and mRNA induced by the acquisition of comorbidity (Fig. 7A, B). It is well-known that the activation of STAT3 signaling induces chromatin remodeling and subsequently enhances the transcription of target genes. The analysis of the GENERADAR and JASPAR databases indicated that the position − 1316/− 1304 in Acp5 promoter contained a potent STAT3 binding site. In PC12 cells, ChIP results revealed that IL-6 incubation increased the binding of p-STAT3 with the Acp5 promoter (Fig. 7C). Moreover, in vivo study showed that the binding of p-STAT3 to the Acp5 promoter in the PrL was significantly enhanced in the comorbidity group (Fig. 7D). To dissect the mechanisms that STAT3 regulates the transcription from the Acp5 promoter, immunoprecipitation (IP) was performed. The results revealed that the interaction between p-STAT3 and P300 was markedly increased in the comorbidity group (Fig. 7E). As P300 plays a critical role in histone acetylation, we further examined whether the histone acetylation levels of the Acp5 promoter region was changed in the comorbidity group. Western blotting results first showed that the total acetylation of H3 (K9) was significantly increased, whereas the levels of acetylated H4 was not changed (Fig. 7F). Next, DNA fragments, extracted from the immunoprecipitation by acetylated H3 antibody, were subjected to PCR to amplify a sequence within the Acp5 promoter region containing the p-STAT3-binding site. The results revealed that the H3 acetylation levels was increased on the Acp5 promoter in the comorbidity group (Fig. 7G). Importantly, the comorbidity-induced acetylated H3 upregulation was decreased by S3I-201 treatment following the acquisition of comorbid in rats (Fig. 7H). These results indicated that STAT3-mediated histone H3 acetylation upregulation at the Acp5 gene promoter contributed to the Acp5 increase in comorbid in rats.

## Discussion

In the present study, we found that rats showed the comorbidity of mechanical allodynia and depression-like behavior on weeks 5 following SNI, and the results of fMRI and electrophysiology indicated that the activity of PrL pyramidal neurons were significantly decreased in the comorbid rats. Furthermore, the Acp5 upregulation in pyramidal neurons, but not PV neurons or SST neurons, mediated the SNI-induced comorbidity symptoms. In mechanism, the activation of IL-6/STAT3 signal pathway, via binding to the promoter region of Acp5, induced the hyperacetylation of histone H3 in promoter region and promoted the expression of Acp5 in PrL pyramidal neurons in the comorbidity rats following SNI. These results suggested that the Acp5 upregulation mediated by the IL-6/STAT3 pathway, via modulating the activity of PrL pyramidal neurons, contributed to the formation of neuropathic pain/depression comorbid induced by SNI in rats.

Numbers studies had proved that mPFC is composed of three brain region: ACC, PrL and IL, and is involved in pain perception, motivational drive, substance seeking and anxiodepressive states [44, 45]. However, whether PrL participated in the neuropathic pain/depression comorbid behavior in rats is largely unclear. Here, we first found that animals exhibited chronic mechanical allodynia and the depression-like behavior including the increased immobility time in FST, the decreased center distance in OFT, the decrease of the spent time and the entries in the open arms in EPM on weeks 5 after SNI, suggested a successful establishment of SNI-induced comorbid symptoms in rats. Moreover, using resting-state fMRI, we found that the fALFF value of PrL was decreased on weeks 5 after SNI relative to the sham group. Evidence showed that the ALFF value during resting state is considered to be physiologically meaningful and reflected the temporal change in neural activity in brain regions [8]. For example, The increase of ALFF indicates the enhanced activity of brain neurons [46]. Our data first suggested that long term nerve injury via reduced the neural activity change the PrL adaptation, which may be associated with the comorbid behavior in rats. It is supported by the notion that adaptive changes in brain contributed to the psychiatric mental disorder [47]. Furthermore, the present electrophysiological result also showed that the excitability of pyramidal neurons in PrL was significantly decreased on weeks 5 after SNI, which are in line with the study that physical restraint stress (PRS) induces the reduction in excitability of pyramidal neurons in PrL in parallel with the development of depression-like behaviors [48]. The decreased activity of PrL pyramidal neurons may result from the suppression of spontaneous activity by continuous nociceptive input [49] or reduced responses to excitatory glutamatergic inputs in pyramidal neurons of PrL [50]. Taken together, the present study showed for the first time that the adaptation of PrL was modulated in the SNI-induced neuropathic pain/depression comorbid symptoms in rats.

Next, we explored the molecular mechanism underlying the decreased excitability of PrL pyramidal neurons. Utilizing bioinformatics analysis and other molecular assays, we found that the Acp5 expression in PrL neurons was significantly increased in the SNI-induced comorbid rats. Genetic knockdown of PrL Acp5 in pyramidal neurons, but not PV neurons or SST neurons, inhibited the decreased excitability of pyramidal neuron and ameliorated comorbid-like behavior induced by SNI in rats. Besides, overexpression of Acp5 in pyramidal neurons reduced the number of action potential and induced the mechanical allodynia and depression-like behavior in naïve rats. These results indicated that the upregulation of Acp5 in pyramidal neurons via regulating the excitability of PrL pyramidal neurons contributed to the neuropathic pain/depression-like symptoms in SNI rats. Studies showed that Acp5, originally as a marker of osteoclasts [51], was also distributed in immune system and nervous system [52, 53]. However, the function of Acp5 in the nervous system has not been reported. Some studies believed that neuropathic pain and depression share some common pathogenetic mechanisms. Therefore, it is hypothesis that the upregulated Acp5 may be a common molecular, via modulated the activity of immune system and nervous system, to contributed to the neuropathic pain and depression-like behavior induced by SNI. The present study is the first report that Acp5 may be involved in the sensory and emotional disorders.

Accumulative evidence indicated that inflammatory responses participated in the neuropathic pain and depression. For example, spinal nerve injury elevates the proinflammatory cytokine IL-1β in the central nervous system including the brainstem, thalamus/striatum and prefrontal cortex [54], and the increases of proinflammatory cytokine such as TNF-α or IL-1β in peripheral or brain are sufficient to produce neuropathic pain or depression [55]. The present study found that the IL-6 expression in PrL was significantly increased on weeks 5 after SNI, and the knockdown of IL-6R in pyramidal neuron of PrL relieved the depression-like behavior induced by nerve injury. These data indicated that the IL-6 upregulation in the PrL pyramidal neurons contributed to the SNI-induced comorbid-like behavior in rats. Moreover, in vivo and in vitro results further showed that the IL-6 receptor mediated the transcriptional factor STAT3 activation on weeks 5 after SNI. STAT3 is an important transcription factor, which regulates the expression of many molecular through binding to the special site of genes [31, 56]. This study revealed that the enhancement of p-STAT3 binding to the Acp5 promoter in PrL, via interaction with P300, induced hyperacetylation of histone H3 and facilitated the transcription of Acp5 in PrL pyramidal neurons. Furthermore, Inhibition of IL-6/STAT3 pathway prevented the Acp5 upregulation through inhibited the p-STAT3-mediated H3 hyperacetylation.

## Conclusions

Taken together, the IL6/STAT3/Acp5 pathway modulated the excitability of PrL pyramidal neurons to change the adaption of PrL region, which critically involved in the development of comorbidity of neuropathic pain/depression. Clinically, the intertwining action between chronic pain and depression substantially perplexes the treatment of neuropsychiatric disorders. Our findings may provide a novel direction from the perspective of comorbidity and identify the potential molecular target for the practical treatment of chronic pain and depression in patients.

## Data Availability

The data sets used during the current study are available from the corresponding author on reasonable request.

## Abbreviations

PrL: Prelimbic cortex
mPFC: Medial prefrontal cortex
fMRI: Functional magnetic resonance imaging
SNI: Spared nerve injury
SD: Sprague–Dawley
AAV: Adeno-associated virus
FST: Forced swimming test
OFT: Open field test
EPM: Elevated plus maze
TR: Repetition time
TE: Echo time
EPI: Echo-planar image
FOV: Field of view
FWHM: Full width half-maximum
fALFF: Fractional amplitude of low-frequency fluctuations
ACSF: Artificial cerebrospinal fluid
rsfMRI: Resting-state fMRI
RMP: Resting membrane potential
